# Extracranial Facial Nerve Schwannoma—Histological Surprise or Therapeutic Planning?

**DOI:** 10.3390/medicina59061167

**Published:** 2023-06-17

**Authors:** Daniela Vrinceanu, Mihai Dumitru, Matei Popa-Cherecheanu, Andreea Nicoleta Marinescu, Oana-Maria Patrascu, Florin Bobirca

**Affiliations:** 1 ENT Department, Carol Davila University of Medicine and Pharmacy, 050472 Bucharest, Romania; vrinceanudana@yahoo.com; 2 Department of Cardiovascular Surgery, “Prof. Dr. Agrippa Ionescu” Emergency Clinical Hospital, 011356 Bucharest, Romania; 3 Radiology Department, Carol Davila University of Medicine and Pharmacy, 020021 Bucharest, Romania; andreea_marinescu2003@yahoo.com; 4 Department of Pathology, Carol Davila University of Medicine and Pharmacy, 050096 Bucharest, Romania; oanamaria.patrascu@gamil.com; 5 Department of Surgery, Carol Davila University of Medicine and Pharmacy, 011437 Bucharest, Romania; florin.bobirca@umfcd.ro

**Keywords:** schwannoma, facial nerve, extracranial, facial palsy, surgery, histology

## Abstract

Schwannomas (neurilemomas) are benign, slow-growing, encapsulated, white, yellow, or pink tumors originating in Schwann cells in the sheaths of cranial nerves or myelinated peripheral nerves. Facial nerve schwannomas (FNS) can form anywhere along the course of the nerve, from the pontocerebellar angle to the terminal branches of the facial nerve. In this article, we propose a review of the specialized literature regarding the diagnostic and therapeutic management of schwannomas of the extracranial segment of the facial nerve, also presenting our experience in this type of rare neurogenic tumor. The clinical exam reveals pretragial swelling or retromandibular swelling, the extrinsic compression of the lateral oropharyngeal wall like a parapharyngeal tumor. The function of the facial nerve is generally preserved due to the eccentric growth of the tumor pushing on the nerve fibers, and the incidence of peripheral facial paralysis in FNSs is described in 20–27% of cases. Magnetic Resonance Imaging (MRI) examination is the gold standard and describes a mass with iso signal to muscle on T1 and hyper signal to muscle on T2 and a characteristic “darts sign.” The most practical differential diagnoses are pleomorphic adenoma of the parotid gland and glossopharyngeal schwannoma. The surgical approach to FNSs requires an experienced surgeon, and radical ablation by extracapsular dissection with preservation of the facial nerve is the gold standard for the cure. The patient’s informed consent is important regarding the diagnosis of schwannoma and the possibility of facial nerve resection with reconstruction. Frozen section intraoperative examination is necessary to rule out malignancy or when sectioning of the facial nerve fibers is necessary. Alternative therapeutic strategies are imaging monitoring or stereotactic radiosurgery. The main factors which are considered during the management are the extension of the tumor, the presence or not of facial palsy, the experience of the surgeon, and the patient’s options.

## 1. Introduction

Schwannomas (neurilemomas) were first described by Virchow in 1908. They are benign, slow-growing, encapsulated, white, yellow, or pink tumors originating in Schwann cells in the sheaths of cranial nerves or myelinated peripheral nerves. Their capsule continues directly with the epinerve. Occasionally, they may show calcifications or cystic degeneration [1].

One in four schwannomas originates in the nerve structures of the head and neck. When located in the head and neck, most schwannomas involve the facial nerve. Facial nerve schwannomas (FNS) can form anywhere along the course of the nerve, from the pontocerebellar angle to the terminal branches of the facial nerve. They are rare tumors, representing less than 1% of schwannomas. Many are located in the intratemporal region, and only 9% involve the extratemporal segment [2].

Of all FNS, approximately 9% occur in the intraparotid segment. Intraparotid FNSs represent 0.2–1.5% of all facial nerve tumors. Caughey et al., in a 38-year retrospective study of FNS involving the parotid gland, found 3722 patients with schwannoma, of whom 29 were FNS and only eight involved the parotid segment of the facial nerve [3].

Since it is a rare type of tumor, it is relatively difficult to standardize diagnostic and therapeutic management. In this article, we propose a review of the specialized literature regarding the diagnostic and therapeutic management of schwannomas of the extracranial segment of the facial nerve. Also, we present our experience with this type of rare neurogenic tumor. Articles in the English language about the management of FNS have been selected and critically reviewed. When we searched for facial nerve schwannoma, we found 3179 results. When we added the extracranial segment, there were 51 results, and when we used intraparotid facial nerve schwannoma, we found 84 results.

## 2. Anatomical Reminder

**Figure 1:**
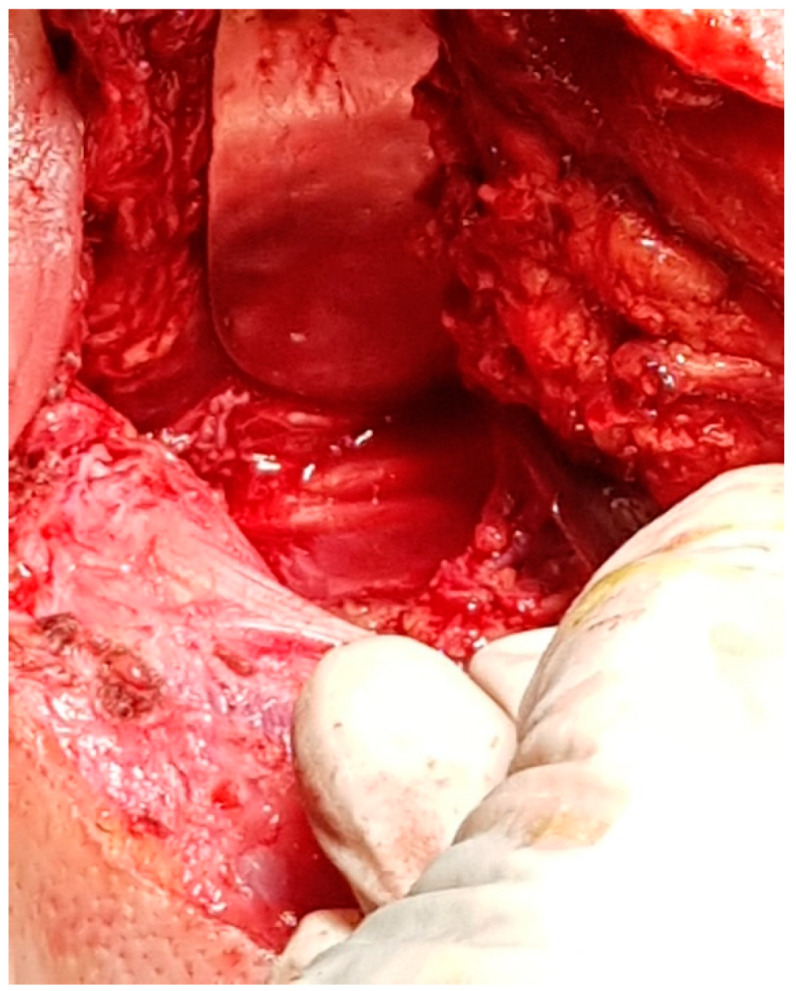
Intraoperative view of the right facial nerve trunk on the external face of the right styloid process, after ablation of a facial nerve schwannoma in the retro parotid segment of the nerve, with preservation of the nerve trunk.

**Figure 2:**
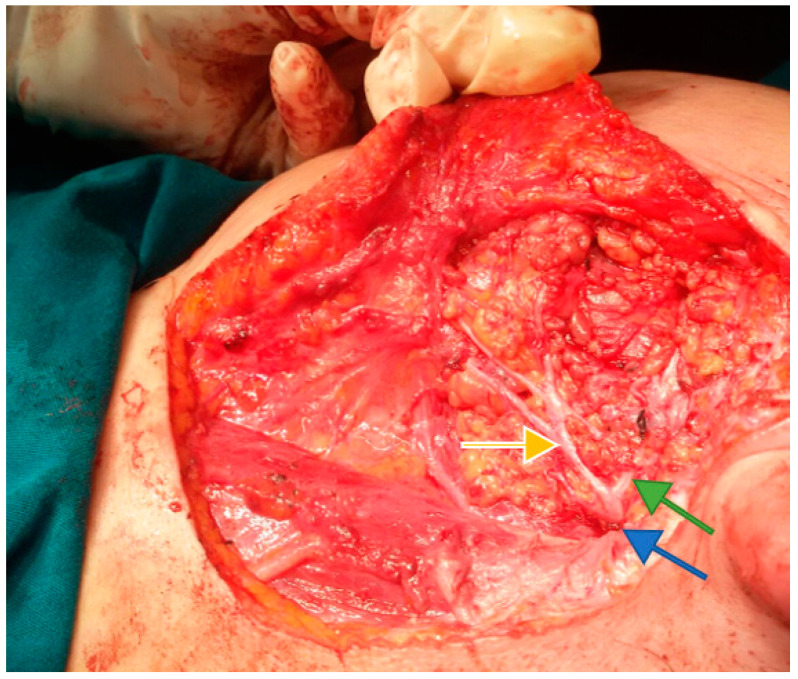
The trunks and branches of the facial nerve, after superficial parotidectomy. Shown here are the trunk of the facial nerve (blue arrow), which bifurcates into the temporal-facial branch (green arrow), and the cervicofacial branch (yellow arrow).

It is important to remember that three distinct segments of the extracranial portion of the facial nerve are described: retro parotid segment, intraparotid segment, and extra parotid segment. The retroparotid segment is the shortest (10–12 mm), but it is the most important from a surgical point of view because it is discovered at this level [4]. It exits the skull through the stylomastoid foramen with an antero-infero-external trajectory. It crosses the external face of the styloid apophysis and the styloid diaphragm between the posterior belly of the digastric (external) and the stylohyoid muscle (internal), finally reaching the parotid gland (Figure 1). The intraparotid segment is approximately 2 cm long and projects to the skin on a line that joins the earlobe to the wing of the nose. This segment has an anterior and external course, becoming shallower at the ramification [5]. It crosses the external carotid artery, passing laterally. Afterward, it encounters the external jugular vein—near the posterior margin of the mandible. There, it classically divides into two terminal branches—the temporofacial branch and the cervicofacial branch (Figure 2). Davis et al. were the first to describe the branching variability of the facial nerve plexus and propose a classification [6]. Last year Poutoglidis et al. published a systematic review of the branching patterns of the facial nerve, concluding that type III is the most common branching pattern instead of type I according to the Davis classification [7]. The complexity of these branching patterns creates a difficult field for the surgeon to orient and dissect the facial nerve inside the parotid gland. Recently immunohistochemistry studies showed that the fibers traveling inside the postparotid terminal cranial nerve VII branch connections are not exclusively motor, and this could explain synkinesis after surgery [8]. The branching pattern of the facial nerve is of extreme importance also in the newer techniques for facial reanimation surgery using the nerve fibers supplying the zygomaticus major muscle [9]. One of the rarest branching patterns is that of double main trunks FN, not mentioned in the Davis classification system [10].

The pharyngeal extension of the parotid gland can extend into the parapharyngeal space and displace the pharyngeal wall when a tumor develops at this level [11]. Schwannomas of the extracranial segment of the facial nerve can extend into the infratemporal fossa. The infratemporal fossa represents an irregular, retro maxillary space, bordered as follows: superiorly by the greater wing of the sphenoid (medial) and by the squama temporal (lateral); medial to the lateral surface of the lateral blade of the pterygoid process; anterior to the posterior wall of the maxillary sinus; inferior has direct communication with the neck and is partially closed by the medial pterygoid muscle [12]. The parapharyngeal space and infratemporal fossa can be occupied by schwannomas developed from the extracranial portion of the facial nerve, especially from the retroparotid portion or the deep parotid lobe [13].

## 3. The Clinical Picture of Extracranial FNSs

**Figure 3:**
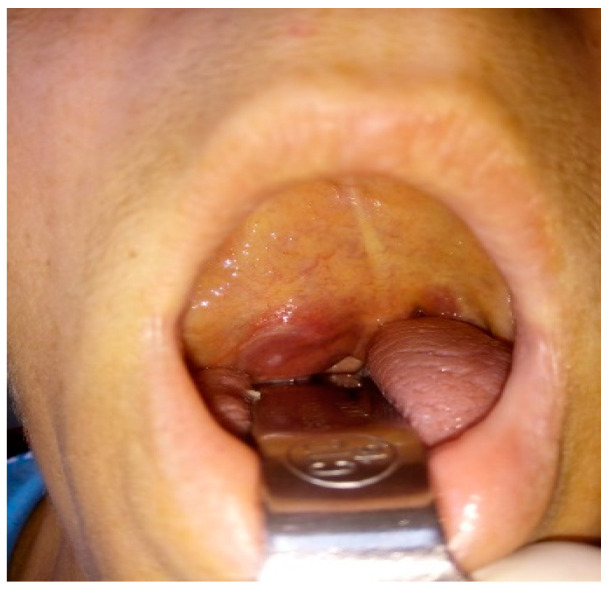
Right parotid deep lobe schwannoma, with clinical presentation of right parapharyngeal tumor, with bulging at the level of the right oropharynx and pushing the right palatine tonsil downward and anteriorly.

**Figure 4:**
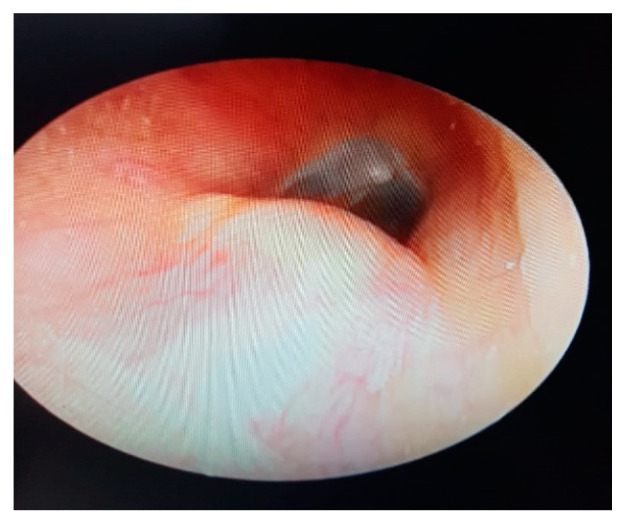
Otic endoscopy view of the right ear in a patient with a right deep parotid lobe tumor pushing and deforming the inferior wall of the right external auditory canal with secondary canal stenosis.

**Figure 5:**
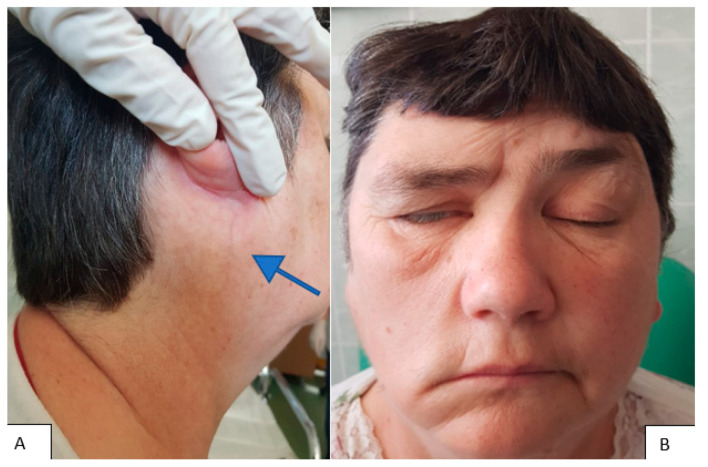
(**A**). Clinical appearance for right deep parotid lobe tumor, with right retroangulomandibular swelling (arrow). (**B**). Right peripheral facial paresis, more than 6 months old.

The clinical picture of the extracranial segment of FNS is uncharacteristic, and only 20% of patients show symptoms because the tumor has slow growth and a late clinical onset [14]. The clinical picture can be represented by a painless preauricular swelling or by a tumor of the deep parotid lobe with a clinical picture like that of a parapharyngeal tumor (Figure 3). In this context, through the development of the tumor in the parapharyngeal space or the infratemporal fossa through the compression of the Eustachian tube, we can clinically encounter a picture of chronic serous otitis [15]. In the variant of FNS that develops at the level of the stylomastoid foramen concerning the trunk of the facial nerve, it is possible to deform the lower wall of the bony external auditory canal (tympanic bone) with canalicular stenosis and hearing loss (Figure 4). Also, the tumor can become, after a certain period of evolution, palpable as retroangulomandibular swelling (Figure 5). Although it is a tumor that develops from the facial nerve sheath, the function of the facial nerve is generally preserved due to the eccentric growth of the tumor pushing on the nerve fibers and, secondarily, the ability of the parotid gland to accommodate the growth of the tumor. The incidence of peripheral facial paralysis in FNS is described in the literature as between 20–27% of cases [16].

## 4. Imagery in Extracranial FNSs 

**Figure 6:**
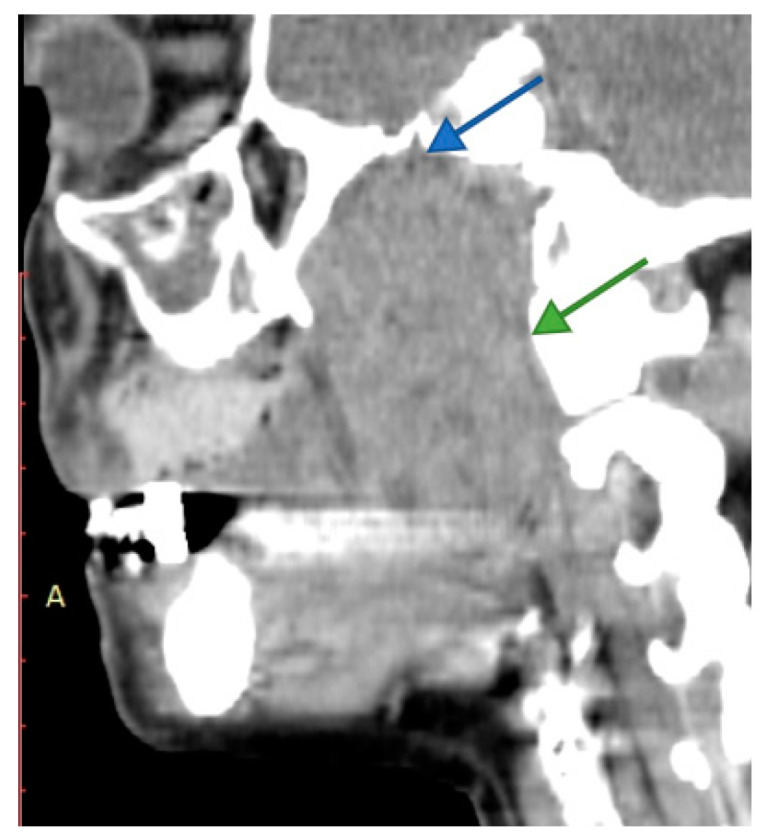
Cervical CT view in the sagittal plane showing an extensive facial nerve schwannoma in the right infratemporal fossa, retro maxillary, with the upper pole in contact with the base of the skull (blue arrow) and the posterior pole in the prevertebral plane (green arrow).

**Figure 7:**
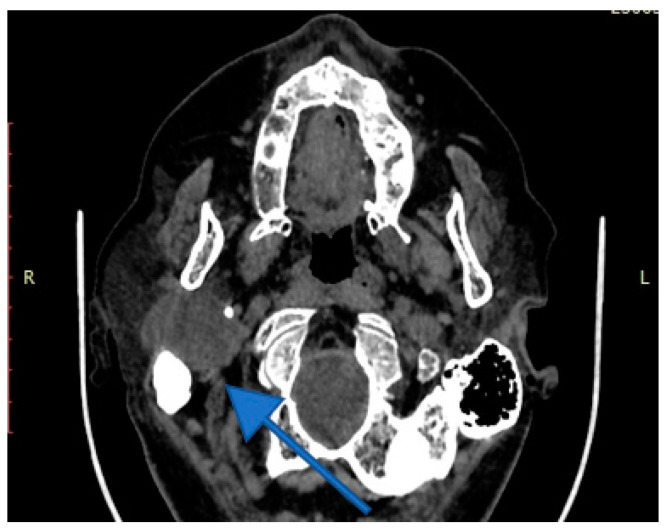
Contrast-enhanced cervical CT view showing deep lobe tumor of the right parotid gland, with extension into the right infratemporal fossa widening the distance between the styloid process and the mastoid process of the temporal bone (arrow).

**Figure 8:**
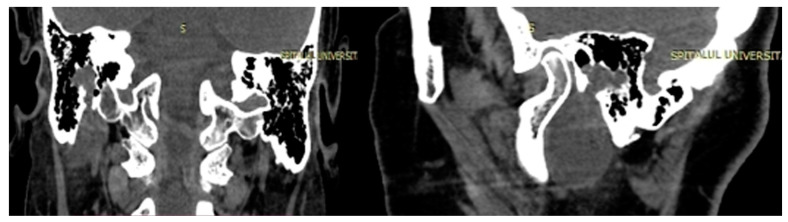
Temporal bone CT showing facial nerve schwannoma developed at the level of the stylomastoid foramen with minimal extension into the mastoid segment of the facial nerve (coronal and sagittal sections).

**Figure 9:**
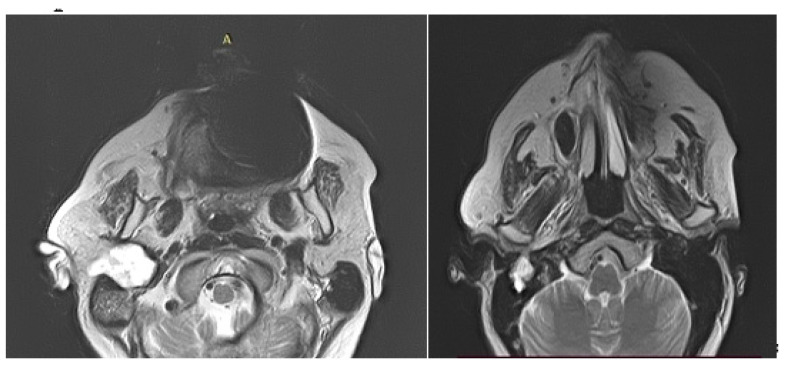
Cervical MRI depicting a deep lobe tumor of the right parotid gland with schwannoma histology after surgery, with extension in the right infratemporal fossa towards the stylomastoid foramen (axial sections).

**Figure 10:**
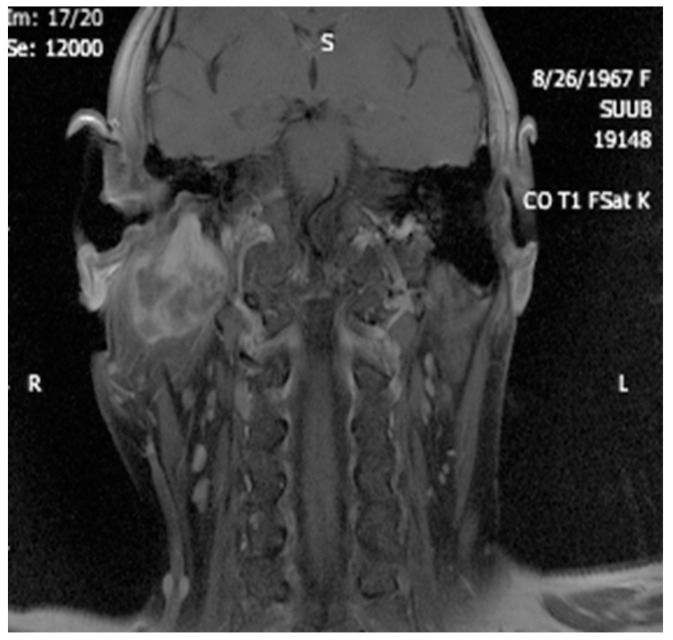
MRI appearance of a nonhomogeneous tumor developed at the level of the right stylomastoid foramen, with an appearance suggestive of a schwannoma of the right facial nerve (coronal section).

**Figure 11:**
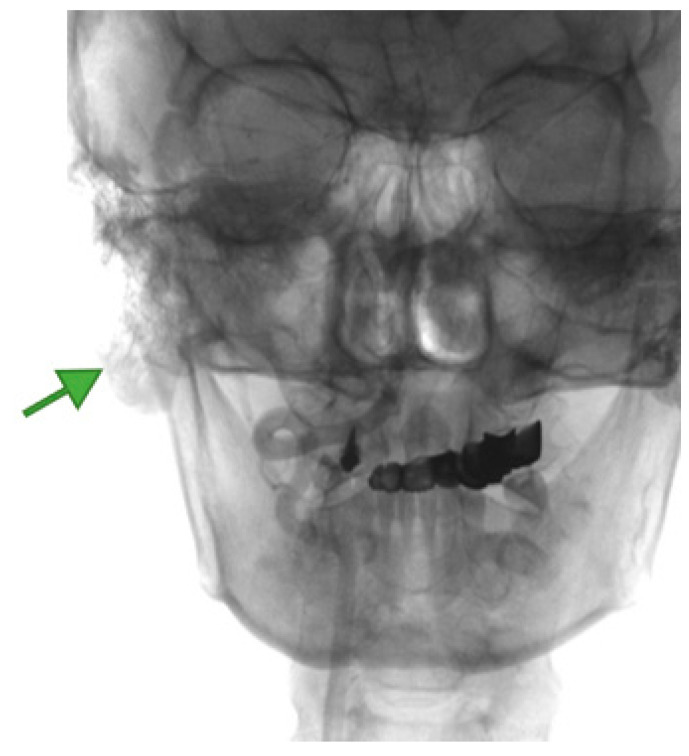
Right carotid angiography, with tumoral blush (green arrow) at the level of a facial nerve schwannoma of the right deep parotid lobe, with the normal angiographic appearance of the carotid system, in the image showing a normal route of the right occipital artery that is in contact with lower tumor pole.

**Figure 12:**
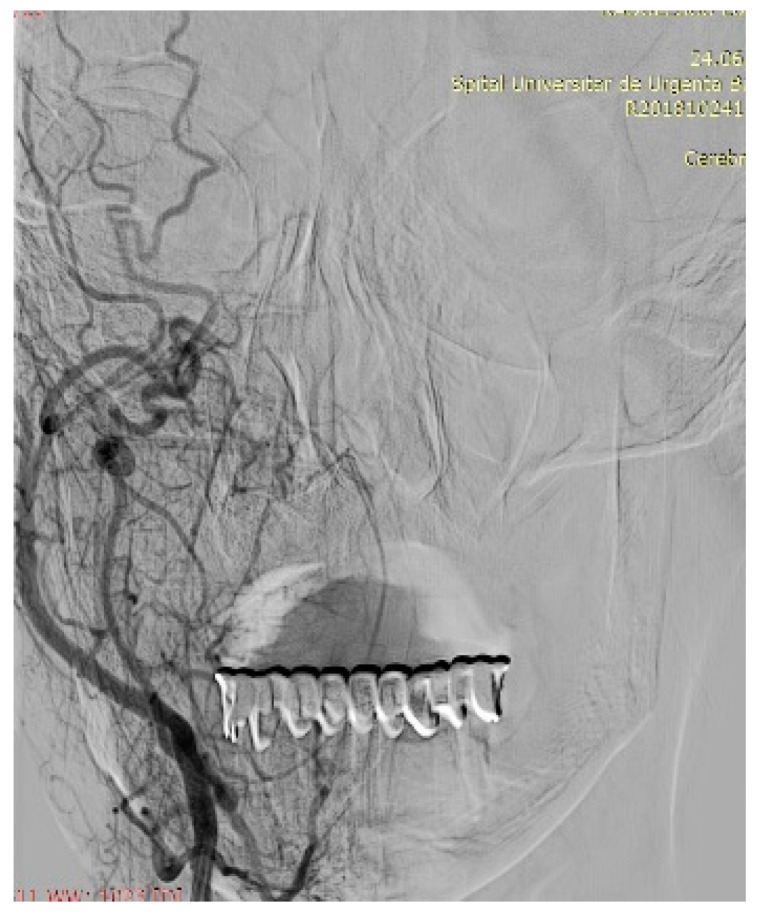
Right carotid angiography with tumor blush in a patient with voluminous facial nerve schwannoma extending into the right infratemporal fossa.

Imaging is essential for therapeutic planning. Contrast-enhanced cervical CT scan shows an inhomogeneous, well-circumscribed lesion and may reveal compression bone lysis lesions (Figure 6 and Figure 7). Detailed information can be provided by the CT scan of the temporal bone, which can highlight FNS development at the level of the stylomastoid foramen with secondary changes induced in the mastoid portion of the facial nerve (Figure 8). MRI examination is the gold standard in the formulation of high suspicion of schwannoma, even before performing any fine needle aspiration cytology (FNAC) maneuver or any surgical exploration [17]. MRI examination describes a mass with iso signal to muscle on T1 and hyper signal to muscle on T2 [18]. The “darts sign” is characteristic, with a weak central signal and high peripheral signal in T2, being suggestive of benign or malignant neurogenic tumors (Figure 9 and Figure 10). Carotid angiography [19] can be useful in large tumors, having an exploratory purpose, and in selected cases, with voluminous tumors, it can also have a therapeutic purpose through preoperative selective embolization (Figure 11 and Figure 12).

Ultrasound examination is insufficient for diagnosis, as well as for preparation for surgery, but it can be useful in raising the suspicion of a tumor of the deep parotid lobe and in the dimensional monitoring of an already detected tumor [20]. FNAC, which is a common procedure to treat tumoral pathology of the parotid gland, does not bring the same benefit in FNS, even performed under ultrasound guidance, being burdened by the risk of damaging the facial nerve threads and inconclusive results which, in any case, do not change the need for surgical exploration and obtaining a significant sample for histological diagnosis [21]. Of course, under the conditions of an experienced surgeon-pathologist team, FNAC can bring significant diagnostic benefits. Essentially, in the absence of histology, preoperative diagnosis is practically impossible if there is only a high suspicion of schwannoma following the MRI examination and especially if there is also clinical peripheral facial palsy.

## 5. Histology

**Figure 13:**
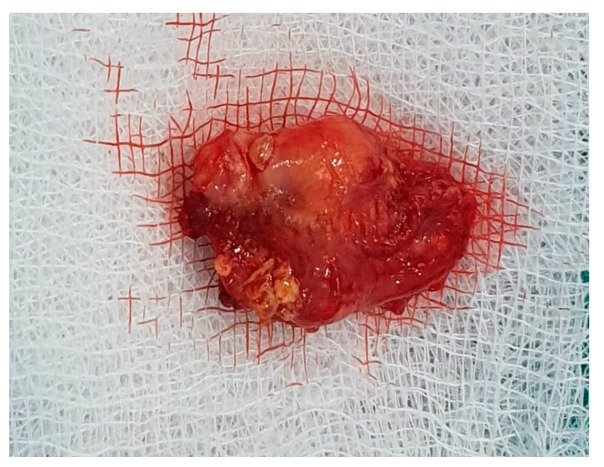
Macroscopic appearance of facial nerve schwannoma: cystic appearance with a thin wall and yellowish friable intraluminal material.

**Figure 14:**
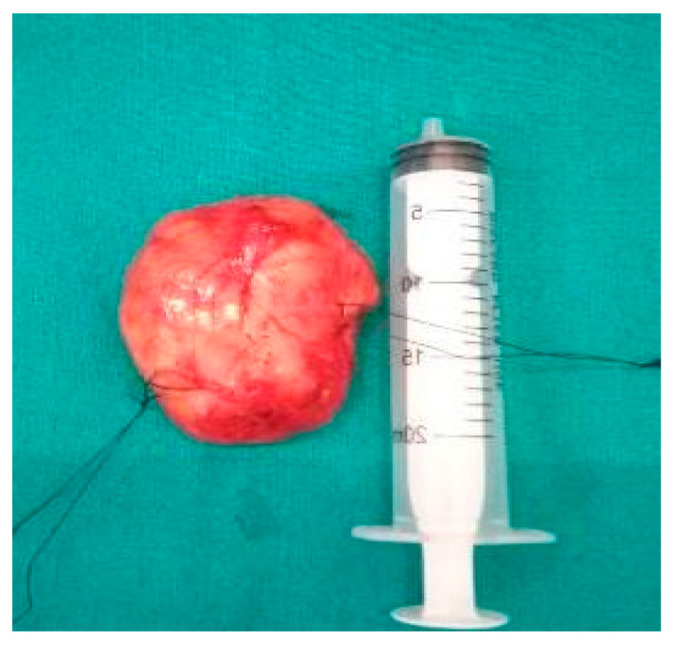
Macroscopic appearance of nodular facial nerve schwannoma.

**Figure 15:**
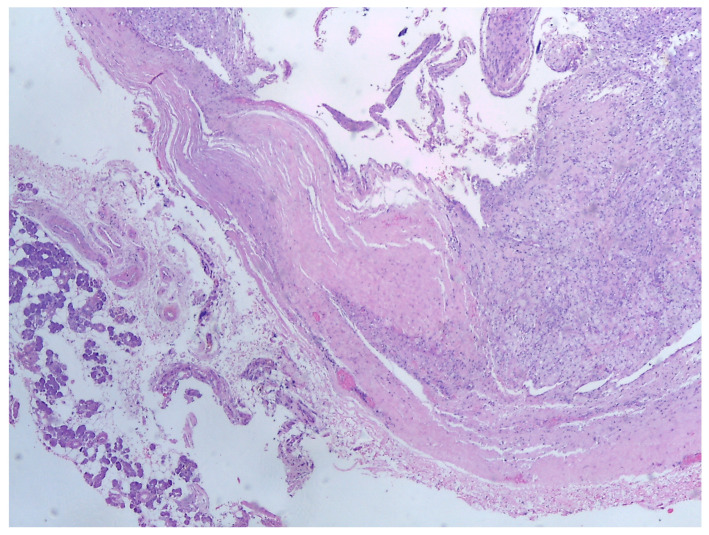
On the left side of the image, there is a glandular tissue of the parotid gland, and on the right side, there is a spindle-cell proliferation with a thick fibrous capsule and a hyalin stroma, HE, 40×.

**Figure 16:**
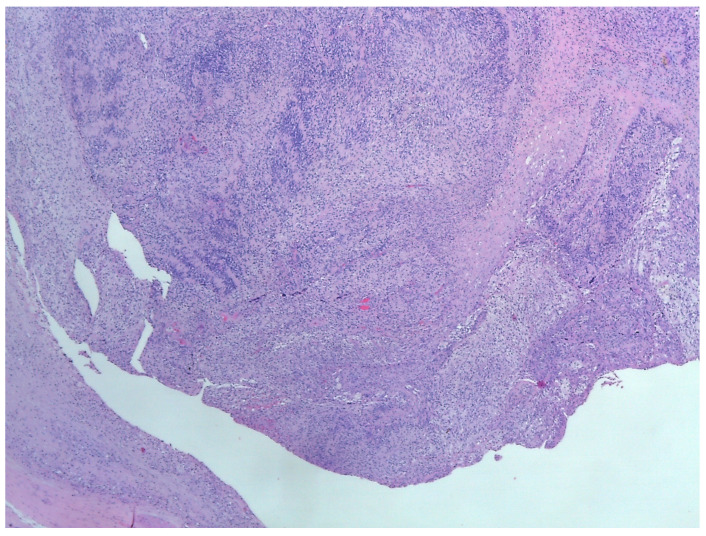
Encapsulated nodular proliferation with spindle cell originating in Schwann cell and cells with clear cytoplasm admixed a fibro-hyalin stroma, HE, 40×.

**Figure 17:**
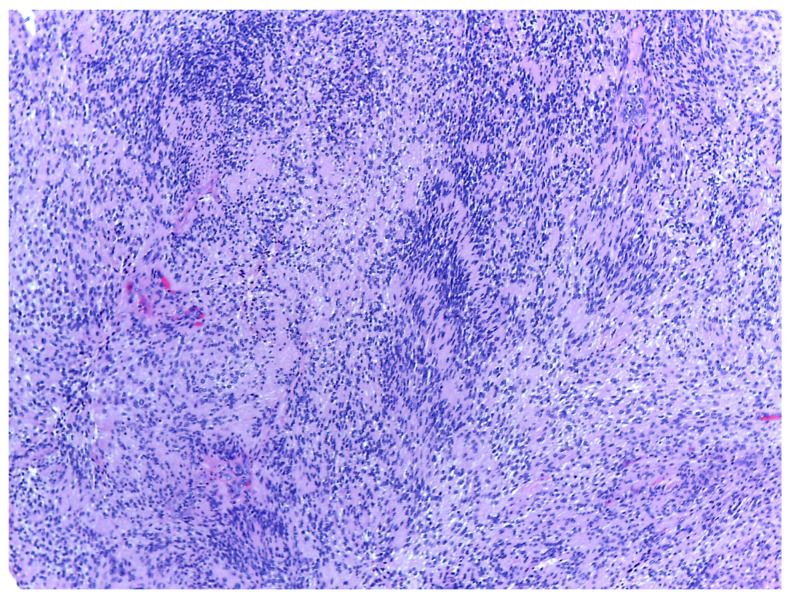
Details of the spindle cell proliferation pattern, with hypercellular Antoni A areas with Verocay bodies (where the nuclei of the cells are arranged in a palisaded architecture); a hyalinized blood vessel can also be seen, HE, 100×.

**Figure 18:**
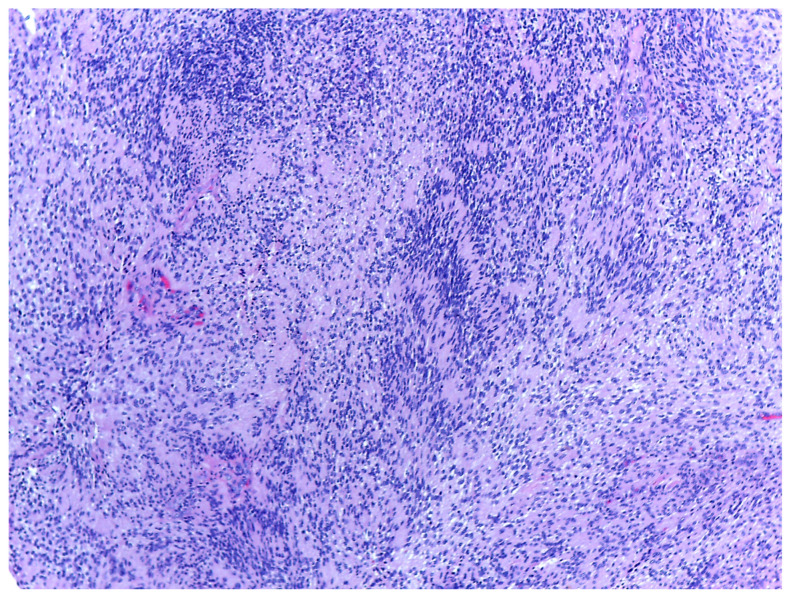
Details of the myxoid area (Antoni B area) and blood vessels with thick hyalinized walls, HE 100×.

Histology is the only one that provides the diagnosis with certainty, the result being, sometimes, surprising for a tumor of the deep parotid lobe. On macroscopic examination, FNSs appear as a well-defined cystic mass with a smooth surface and a variety of colors (yellowish, pinkish, grayish-white, reddish) and, in some cases, lobulated (Figure 13 and Figure 14). From a histological point of view, two types of areas are described in FNS: Antoni A areas are hypercellular areas with elongated, fusiform cells, with nuclei aligned in the palisade and Verocay bodies (acellular zones, located between the nuclear palisades) and Antoni B areas are hypocellular, with cellular pleiomorphism, without a palisade aspect for the nuclei [22]. Both types of areas are found in different proportions in FNS (Figure 15 and Figure 16). Nerve fibers (axons of the facial nerve) are not part of the tumor, recalling that the tumor develops from the sheath of Schwann and pushes the axons to one side (Figure 17 and Figure 18). The histological paraffin examination should be completed with immunohistochemistry for the S100 marker—necessary to establish the neural origin, and for SMA (Smooth Muscle Actin)—to exclude a leiomyoma [23].

## 6. Differential Diagnosis of the Extracranial FNS

The differential diagnosis must be made, most often, with a pleomorphic adenoma of the parotid gland that is difficult to differentiate from imaging studies such as CT and MRI [24]. FNAC does not provide conclusive results, so a definite diagnosis is possible only after surgical ablation and histological and immunohistochemical examinations. Schwannoma-like pleomorphic adenoma, a rare histopathological variant of pleiomorphic adenoma, is also described [25]. Leiomyoma is another variant of histological diagnosis [26]. For FNS located at the level of the deep parotid lobe, the differential diagnosis of schwannoma of the glossopharyngeal nerve can also be considered [27]. Other differential diagnoses that can be considered include the fluctuating parotid cyst, the lymphoepithelial cyst in HIV-positive patients, and autoimmune diseases that usually cause bilateral lesions and frequently have systemic manifestations [28]. Neurofibromatosis can associate neurofibromas along the facial nerve, but they are usually accompanied by facial paralysis associated with other neurological deficits depending on the location of the neurofibromas, burden, and the risk of recurrence after ablation [29].

## 7. Therapeutic Strategies in the Management of FNS Extracranial Segment

The gold standard treatment of FNS of the parotid segment consists of the surgical excision of the tumor with preservation of the facial nerve. Because these tumors are most often without preoperative peripheral facial palsy, there is a very high moral and medico-legal liability for the surgeon, and it is a fine line between choosing conservatory treatment versus surgical ablation, given the risk of intraoperative damage to the facial nerve [30]. It is possible that in certain selected cases where the imaging arguments are strong in favor of the diagnosis of schwannoma and there is no clinical peripheral facial palsy, the conservative attitude of imaging monitoring of the tumor for a short time may be considered. But, considering that the diagnosis of certainty cannot be formulated by histological examination, and the increase in the size of the tumor also increases the surgical risks, the wisest attitude seems to be informed consent of the patient regarding the operative risks, with the presentation of the possibility of intraoperative reconstruction of a damaged facial nerve [31]. As a consequence, choosing surgery depends on the location and extension of the tumor, the preoperative function of the facial nerve, the experience of the surgeon, and the results obtained previously regarding the reconstruction of the nerve, but also on the patient’s preferences. Large tumors with extension into the mastoid cavity and with preoperative facial paralysis are indications for surgical intervention. An approach to the infratemporal fossa, parapharyngeal space, or parotid gland may be considered [32]. 

**Figure 19:**
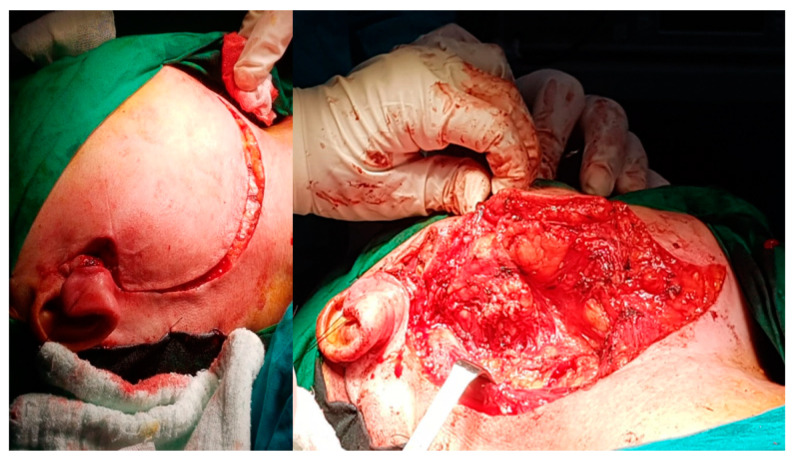
Modified Redon-type cervical parotid approach for deep parotid lobe tumor with the histological result of schwannoma.

**Figure 20:**
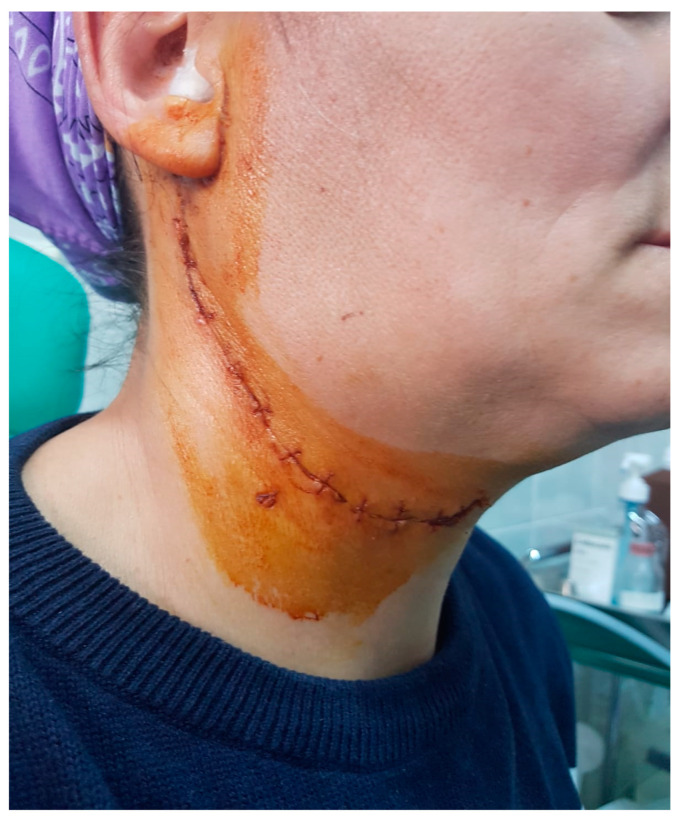
Submandibular arciform approach for right facial nerve schwannoma, from the tip of the mastoid to under the chin, with the steepest point near the hyoid bone in a patient without preoperative peripheral facial palsy.

**Figure 21:**
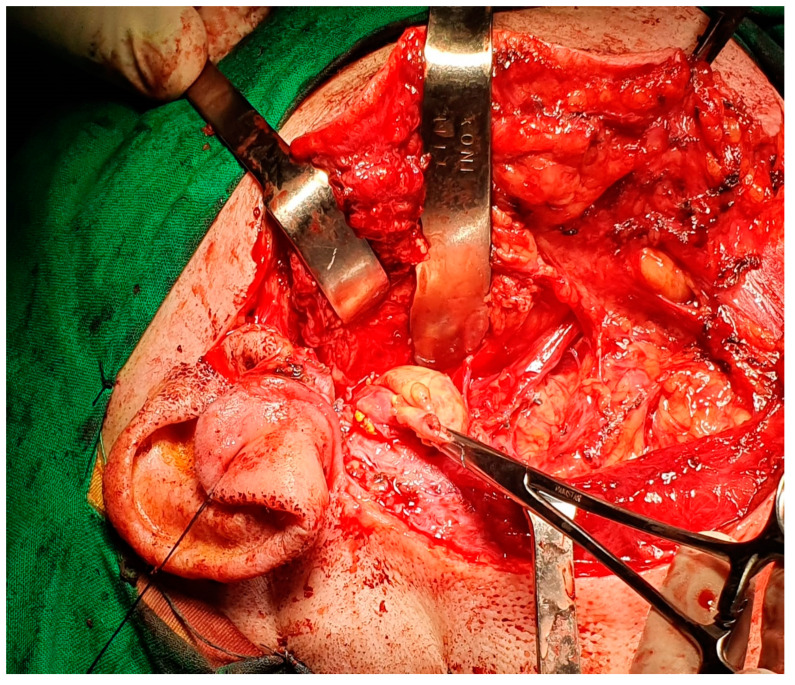
Intraoperative appearance of schwannoma of the facial nerve developed from the retro parotid segment of the facial nerve, with the characteristic yellowish appearance. The right parotid gland is under the retractors.

**Figure 22:**
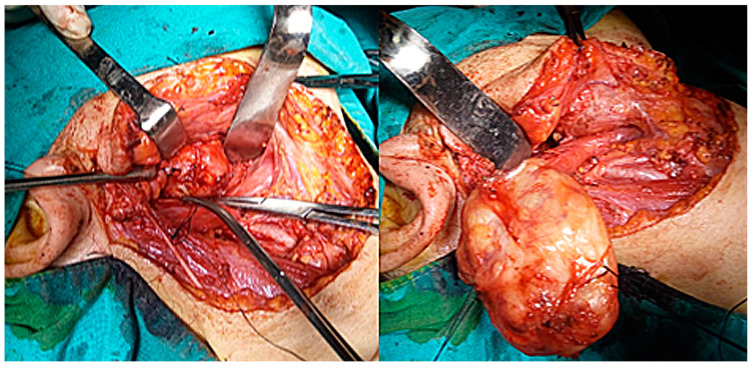
Intraoperative appearance of schwannoma of the facial nerve, of the right deep parotid lobe, with right parapharyngeal evolution, operated using the right submandibular arciform approach—the tumor was removed by extracapsular dissection with preservation of the facial nerve.

**Figure 23:**
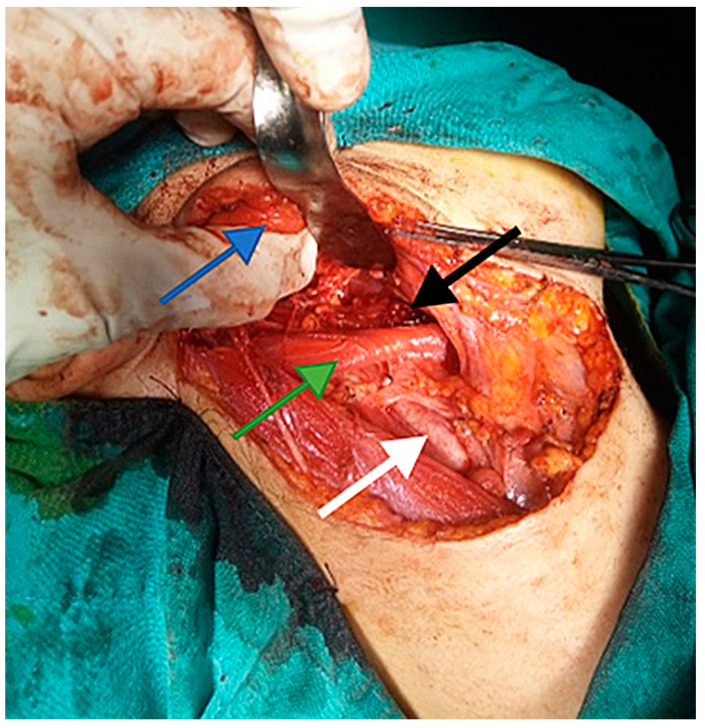
Intraoperative appearance after ablation of a bulky schwannoma of the right deep parotid lobe, with the parotid gland digitally retracted (blue arrow), the posterior belly of the digastric (green arrow), the common carotid artery at the bifurcation (white arrow) and the right parapharyngeal space (black arrow).

The current approach is an arciform submandibular cervical approach or a modified Redon approach (Figure 19 and Figure 20). Tumor ablation by extracapsular dissection with preservation of the facial nerve is ideal. Superficial or total parotidectomy with preservation of the facial nerve can also be considered. In tumors involving the medial aspect of the deep lobe, parotidectomy is usually not necessary (Figure 21, Figure 22 and Figure 23). Frozen sections examination must be done intraoperatively to rule out malignancy, especially if sectioning of the facial nerve fibers is required.

Marchioni D. et al. classify intraparotid FNSs [33] according to the relationship with the facial nerve path into four types of SNF: type A, in which the tumor can be resected without sacrificing the facial nerve; this type of tumor rarely produces preoperative facial paralysis; type B, in which the tumor can be resected, but with a partial sacrifice of peripheral branches or distal divisions; immediate reconstruction, using either a nerve graft or direct neurorrhaphy, is required, and the prognosis is dependent on the affected branch rather than the type of reconstruction; type C, in which resection of the tumor requires the sacrifice of the trunk of the facial nerve; and type D, in which tumor resection requires the sacrifice of the facial nerve trunk and at least one of the temporofacial or cervicofacial branches.

**Figure 24:**
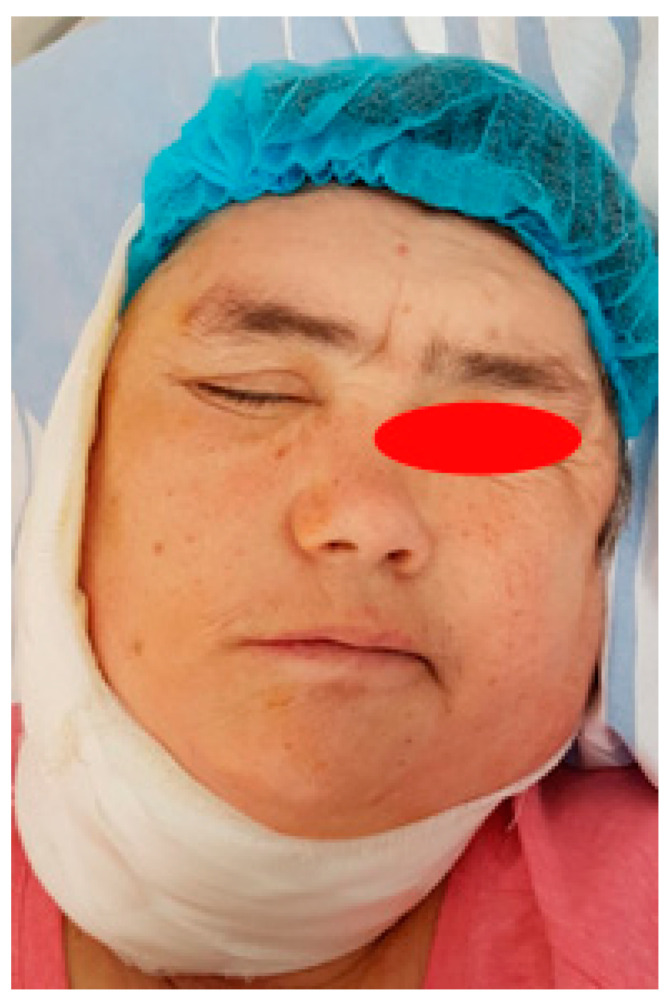
Immediate postoperative appearance of a patient with preoperative right peripheral facial weakness, in whom the facial nerve was preserved, with possible right palpebral occlusion.

**Figure 25:**
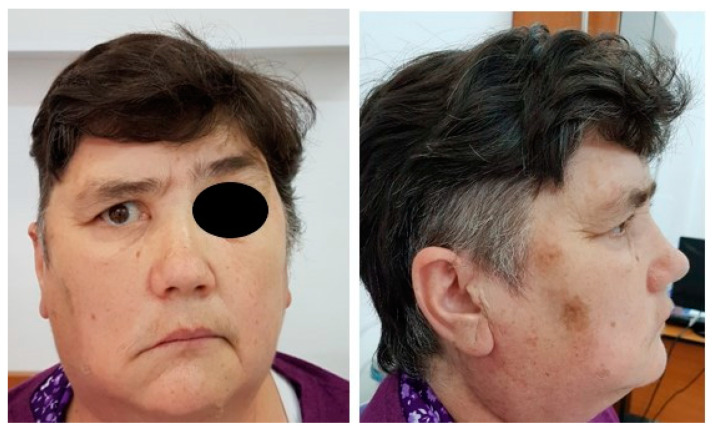
Postoperative appearance at 30 days in a patient with schwannoma of the right facial nerve, parotid segment, with preoperative right peripheral facial paresis older than 6 months, where the right facial nerve was preserved, but in which motor recovery was only partially achieved due to the age of the compressed nerve fibers.

Half of the intraparotid segment FNSs are described in the literature as originating in the trunk of the facial nerve, making it virtually impossible to discover the trunk. Peripheral facial paralysis is reported in intraparotid NSF in which only biopsy or even resection was performed with apparent preservation of the nerve due to the individual sensitivity of the facial nerve to dissection and the particularities of nerve microvascularization [34]. Therefore, even in the conditions of preservation of the facial nerve, after tumor ablation, a reversible peripheral facial paresis is possible under cortisone treatment and group B vitamin therapy (Figure 24). Facial gymnastics initiated immediately postoperatively is essential for motor recovery as quickly as possible [35]. In the case of FNSs with preoperative peripheral facial paresis, the recovery of the facial motor deficit, in the conditions of preservation of the facial nerve, is very unlikely; even in these cases, it is worth starting facial gymnastics immediately postoperatively and chronic treatment with neurotrophic drugs for 60 days (Figure 25).

A therapeutic alternative worth considering in the case of extracranial FNS is stereotactic hyper-fractionated radiosurgery which can be chosen as a therapeutic option when the surgical planning offers few chances of preserving the facial nerve when there is already peripheral facial paralysis, and we still aim to preserve the motor function of the facial nerve [36]. The literature also mentions subtotal excision, with annual radiological follow-up, in cases where dissection of the nerve tumor is difficult, but—in our experience—this therapeutic option only prolongs or postpones the moment of a definitive, radical decision and greatly increases the risks of facial nerve sacrifice in revision surgery.

## 8. Discussions

Because extracranial FNS is a rare neurogenic tumor, however, there is no firm consensus regarding the management of the different types of extracranial FNSs. The therapeutic strategy in extracranial FNSs is done keeping account of the location and extension of the tumor, the preoperative function of the facial nerve, the experience of the surgeon regarding cervical surgery, and the reconstruction of the facial nerve, but also the patient’s options [37]. However, the surgical procedures are extensive and pose a great risk to the overall prognosis of the patient, given the possible associated pathologies such as cardiovascular disease, bleeding disorders, or allergic reactions [38]. In current practice, the surgeon approaching a tumor which can be an extracranial FNS has to keep in mind a type of protocol based on these factors. In the type A or B of FNSs (after Marchioni Classification), or in case of a pre-operative facial nerve palsy House-Brackmann (HB) grade IV or worse, the surgery with ablation and if it is necessary, the reconstruction of the nerve could be the reasonable strategy [39]. In the case of pre-operative facial nerve palsy HB grade III or better and type C or D of FNSs (after Marchioni classification), the biopsy is to exclude a malignant variant and conservative management with imaging monitoring every 12 months [40]. 

## 9. Conclusions

Schwannomas of the extracranial segment, particularly of the parotid segment of the facial nerve, are rare tumors that require careful diagnostic and therapeutic management. MRI and CT Scan imaging is essential, often providing complementary information and formulating a diagnosis of high suspicion for schwannoma and allowing planning for surgery. The surgical approach to FNSs requires an experienced surgeon, and radical ablation by extracapsular dissection with preservation of the facial nerve is the gold standard. The patient’s informed consent is important regarding the diagnosis of schwannoma and the possibility of facial nerve resection with reconstruction. Extemporaneous histological examination is mandatory to rule out malignancy or when sectioning of the facial nerve fibers is necessary. The histological examination, which can sometimes be a surprise, must be completed with immunohistochemistry to confirm the diagnosis of schwannoma. Alternative therapeutic strategies are imaging monitoring or stereotactic radiosurgery. The main factors which are influencing the management are the extension of the tumor, the presence or not of facial palsy, the experience of the surgeon, and the patient’s options.

## Data Availability

All data are available from the corresponding authors on request.
